# Assessing the Current Market of Sunscreen: A Cross-Sectional Study of Sunscreen Availability in Three Metropolitan Counties in the United States

**DOI:** 10.1155/2014/285357

**Published:** 2014-05-15

**Authors:** Kyle T. Amber, Romi Bloom, Patrick Staropoli, Sonam Dhiman, Shasa Hu

**Affiliations:** Department of Dermatology & Cutaneous Surgery, University of Miami Miller School of Medicine, 10660 SW 75th Avenue, Miami, FL 33156, USA

## Abstract

Sunscreen use is recommended for the prevention of sunburn and skin cancer. Little is known regarding sunscreen availability in high versus low income communities. We analyzed sunscreen availability in three large metropolitan counties to determine the relationship between availability and community demographics. We included sun care products in all pharmacies and supermarkets open as of July 2013 in representative high and low income zip codes in Cook County, Illinois, Miami-Dade County, Florida, and San Diego County, California. We recorded the percentage of tanning oil, sunscreens with a sun protection factor (SPF) < 15, SPF > 15, physical sunscreens, spray sunscreens, mean price per ounce (PPO), and mean SPF. Of the total products assessed, 11.0% were tanning oils, with physical sunscreens accounting for only 3.4% of the available sunscreens and 46.2% of sunscreens being spray-on. A comparison between higher and lower income zip codes demonstrated a significantly increased percentage of sunscreens with SPF < 15 in high income zip codes. Lower income zip codes had higher percentages of sunscreens with SPF > 15 and higher PPO, even when taking into account SPF. Further studies of sunscreen usage patterns in different populations must take into account sunscreen availability and price, as these significantly differ based on the community demographic.

## 1. Introduction

Both melanoma and nonmelanoma skin cancers (NMSC) are becoming increasingly prevalent [1, 2], with NMSC becoming the fifth most costly cancer in the United States [3]. Sunburn also accounts for a significant amount of lost work hours, resulting in an estimated economic impact for lost work and treatment in excess of $10 million [4]. Preventive measures against skin cancers and sunburn include sun avoidance during the peak hours, wearing protective clothing, and using sunscreen [5].

Broad spectrum sunscreen leads to a reduction in the formation of thymine dimers* in vivo* [6]. Routine use of sunscreen can prevent the development of NMSC [7, 8]. High sun protection factor (SPF) sunscreens have not been associated with increased sun exposure time in the general population [9]. Additionally, though controversial, regular sunscreen use may prevent the development of melanoma as well as nevi in fair-skinned children [10–12].

Sunscreen use habits can vary by region, education level, socioeconomic status (SES), racial/ethnic background, and age. Coups et al. found that young, Midwestern, non-Hispanic white (NHW), and less-educated males had the greatest number of skin cancer risk behaviors [13]. Buller et al., however, demonstrated that NHW, particularly males with higher incomes [14], experience higher rates of sunburn than Hispanic white (HW) [15]. Among Hispanics, acculturation appears to be positively associated with sunscreen use, with education level being a significant confounding variable [16].

Although the use of sunscreen for skin cancer prevention has been a topic heavily discussed in the media [17], there is little attention paid to the market availability of sunscreen and its possible effect on sunscreen usage. A study of female athletes demonstrated that the application of sunscreen significantly increased with improved access to sunscreen [18]. The cost of sunscreen, especially in the setting of daily use, can be expensive and unmanageable for certain patients who require daily protection [19]. Sunscreen provided to study participants gratis correlated with increased usage with a decrease in sunburn, without an increase in sun exposure time [20]. Thus, the effect of cost on sunscreen usage could perhaps account for the finding that patients of lower SES, especially Hispanics, have a relatively larger proportion of nodular melanomas and relatively fewer superficial spreading melanomas [21].

Though the quality of available sunscreens has significantly improved over the course of the last 15 years, there is little data regarding the availability of quality sun protection in different communities. Wang et al. found marked improvements in sunscreen availability from 1997 to 2009 in the city of Cincinnati, with increases in SPF values and broad spectrum coverage, and a decrease in products with an SPF less than 15 [22]. As sunscreen availability likely varies by geographic location and local demographic characteristics, we sought to compare the availability of sunscreens in three large metropolitan counties in the USA and examine the relationship between sunscreen availability and community demographic characteristics.

## 2. Methods

### 2.1. Study Sites

**Figure 1:**
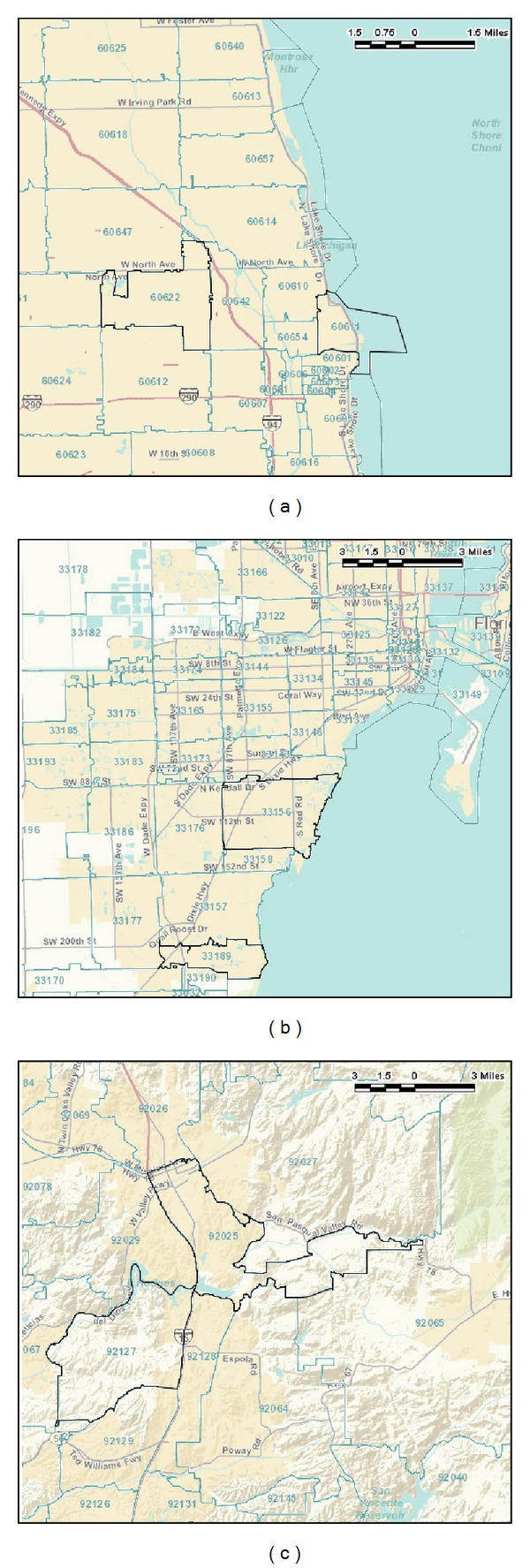
Selected zip codes in (a) Cook County, (b) Miami-Dade County, and (c) San Diego County.

We performed a cross-sectional study to evaluate sunscreens sold at pharmacies and supermarkets in Cook County in Illinois, San Diego County in California, and Miami-Dade County in Florida. Two zip codes, one with high income and the other with low income, were chosen from each county, based on the rank order provided by the U.S. Census [23]. To create a comparable match between the zip codes within each county, we chose zip codes of similar population size and geographic proximity such that the zip codes are not farther than 2 zip codes away from each other. Zip codes 92127 and 92025 (Cook County), 60611 and 60622 (San Diego County), and 33156 and 33189 (Miami-Dade County) were selected. The 2010 U.S. Census data, melanoma incidence [24], and geography of these zip codes are provided in Tables 1 and 2 and Figure 1, respectively.

We evaluated all pharmacies and supermarkets containing a sunscreen or sun care isle in the selected zip codes open as of July 2013. When more than one store of the same chain existed within the zip code, we randomly chose a single store location to include in the study. We evaluated products sold in the sunscreen or sun product isles only. Sunscreens were counted based on their visibility to a consumer. The depth of the shelf was not recorded, but the width of the shelf was; for example, 3 identical sunscreens lined side-by-side would be counted as 3.

### 2.2. Measures

We quantified the market availability of sunscreen products by the percentage of tanning oil, sunscreens with an SPF less than 15 (SPF < 15), sunscreens with an SPF greater than 15 (SPF > 15), physical sunscreens, spray sunscreens, mean price per ounce, and mean SPF. As an SPF > 15 with proportional UV-A coverage has been recommended as a minimum requirement for sunscreens [25], our main outcome measures were the percentages of sunscreens with an SPF > 15, SPF < 15, and the price per ounce of each sunscreen between high and low income zip codes. Secondary measures included the percentage of tanning oils, physical blockers, spray sunscreens, and mean SPF between these high and low income zip codes. With the FDA proposing a cut-off at an SPF of 50+, we calculated a corrected SPF where original values greater than 50 were recorded as 50 [26]. Physical blockers were defined as those sunscreens containing zinc oxide and titanium dioxide. Sunscreens containing chemical UV absorbers or only the physical blocker titanium were considered to be chemical sunscreens, as these lack physical UV-A protection [27].

### 2.3. Statistical Analyses

We performed statistical analysis using the SPSS software version 21 (IBM Corporation, Chicago). To compare the percentage of sunscreens with SPF > 15, SPF < 15, tanning oils, physical blockers, and spray sunscreens between high and low income zip codes, we constructed a 2 × 2 contingency table and performed a *χ* ^2^ analysis. For tests failing to meet the *χ* ^2^ requirements, we used the Fischer-exact test. To compare price per ounce, price, and mean SPF we used an independent-samples* t*-test without the assumption of equal variances. All tests were two sided and significance was defined at the level of *P* < 0.05. A subgroup analysis was performed to evaluate for variations in each geographic location.

## 3. Results

A total of 1,660 sun products were assessed. Tanning oils accounted for 11.0% of total sun products. The percentages of products with SPF < 15 and SPF > 15 were 14.9% and 65.4%, respectively. Physical sunscreens accounted for 3.4% of available sunscreens and were significantly more expensive than chemical sunscreens. 46.2% of sunscreen were spray-on. Spray-on sunscreens were significantly cheaper than lotion sunscreens. The mean price per ounce of sunscreen was $2.10 with a mean SPF of 34.3. Chemical sunscreens and spray sunscreens were significantly cheaper than physical and lotion sunscreens, respectively (Table 3).

A comparison between higher and lower income zip codes demonstrated a significantly increased percentage of tanning oils and sunscreens with SPF < 15 in high income zip codes. Lower income zip codes had higher percentages of sunscreens with SPF > 15 and spray sunscreens, as well as a higher mean corrected SPF (Table 4). The price of sunscreen per ounce was significantly higher in the lower income zip codes than in the higher income zip codes. When price per ounce was adjusted with corrected SPF, the mean price per SPF ounce was still higher than in the lower income zip codes (0.094 versus 0.076, *P* < 0.01). There was no significant difference in the percentage of physical sunscreens among high and low income zip codes.

Subgroup analysis found similar stratification in sunscreen SPF and price only in Miami-Dade County. San Diego County demonstrated similar findings, except that there was no statistically significant difference in the percentage of sunscreens with an SPF > 15 or spray sunscreens. There was no statistically significant difference between the selected high and low income zip codes in Cook County.

### 3.1. Comment

We performed a cross-sectional study of sunscreens available in a representative high and low income zip codes in three major metropolitan counties in the United States. In the 3 counties surveyed, chemical sunscreens and spray sunscreens were more prevalent and inexpensive than physical sunscreens and sun lotions. Sunscreens sampled from San Diego County, the county with the highest incidence of melanoma amongst our sampled counties, had the lowest percentage of tanning oils (5.4%) yet the highest mean price per ounce ($2.22). In contrast, Miami-Dade County, the county with the lowest incidence of melanoma amongst our sampled counties, had the highest percentage of tanning oil availability (18.5%), but the lowest price per ounce of sunscreen ($1.91). From the three sampled counties, tanning oil availability appears to have a negative association with melanoma incidence while the price of sunscreen appears to have a positive association. Amongst all three counties sampled, lower income zip codes demonstrated an increased availability of sunscreens SPF > 15 with a smaller proportion of tanning oils. Prices were, however, higher for sunscreens in lower income zip codes than in higher income zip codes.

### 3.2. Physical versus Chemical Blocker

Sunscreens containing physical UV-A blockers were a lot less available compared to chemical sunscreens (3.4% versus 96.6%) in our study. Chemical sunscreens have certain limitations which makes the apparent lower market share of physical blockers concerning. Butyl methoxydibenzoylmethane (avobenzone) provides UV-A coverage in chemical sunscreens. However, it requires additional chemicals to increase its stability in sunlight [28, 29]. Without these photostabilizers or with the exhaustion of these stabilizers, UV-A protection can significantly decrease in as little as 60 minutes [30, 31]. This was confirmed in a study that demonstrated poor photostability of avobenzone in a majority of sunscreens in the market [32]. Additionally, chemical sunscreen is one of the most common causes of photoallergy [33, 34].

As opposed to chemical sunscreens that provide protection by mainly absorbing UV radiation, physical blockers absorb, reflect, and scatter UV radiation [35]. Physical blocks have superior photostability and are thus recommended for children and sunscreen allergic patients [36]. They are also recommended for use in patients with photoreactions towards visible light [37]. However, users may prefer chemical over physical blockers due to the thicker consistency of physical blocks [38]. This was similarly demonstrated in a study of facial sunscreen vehicles and usage patterns [39]. The significantly lower price per ounce of chemical blockers may additionally lead to increased availability.

### 3.3. Spray Sunscreens

Sunscreens dispensed by spray accounted for 46.2% of sampled sunscreen. The significantly lower price per ounce of spray-on sunscreen may account for its high availability. While spray-on sunscreens may account for a notable proportion of the sun care isle, little data is available regarding the actual amount of sunscreen successfully applied when using a spray sunscreen. Other delivery systems such as a lotion dispenser or stick have been shown to affect the amount of sunscreen users apply [40]. As sunscreen is already typically applied at far less than the thickness required to achieve the labeled SPF [41, 42], more data is needed on the efficacy of spray sunscreen. Additionally, there have been recent concerns regarding the risk of combustion and injury when using these sunscreens [43].

### 3.4. Differences in Sunscreens Offered by Zip Code

Though market availability of sunscreen is insufficient to conclude consumer habits and preventative behaviors, clear differences existed between sunscreen availability in high and low income zip codes overall as well as within Miami-Dade County and San Diego County. Tanning oil and sunscreens with SPF < 15 accounted for a greater proportion of sunscreens sold in the higher income, more educated zip codes, which notably had a higher mean age than their lower income and less educated counterparts. This is alarming given the higher sunburn prevalence in the higher income subset of non-Hispanic males [14, 44]. The relationship between sunburn prevalence and market availability of sunscreen SPF > 15 is not known and may be driven by consumer preference and sun protective behavior, but clearly the higher market share of SPF < 15 sunscreens in communities with higher levels of education and older communities needs to be addressed. As these higher income and more educated zip codes contain a larger percentage of non-Hispanics, it is possible that this is the determinate factor. Additionally, despite an increase of skin cancer risk factors in Midwesterners [13], our study notably demonstrated the lowest proportion of tanning oils and highest proportion of SPF > 15 sunscreens in Cook County provided the best coverage. Tanning oil availability does not appear from our limited sample, however, to be positively associated with melanoma incidence.

Perhaps the most concerning finding is the difference in pricing of sunscreen between high and low income zip codes. Even after correcting for the level of SPF in the sunscreen, lower income zip codes demonstrated a significantly elevated price of sunscreen per ounce. As price can often serve as a deterrent to sunscreen usage [19], this effect could be magnified in a low income community. Additionally, as price was the highest in the county with the greatest melanoma incidence and lowest in the county with the lowest melanoma incidence, further research is needed regarding the association between sunscreen prices and skin cancer.

### 3.5. Limitations

The use of a single high income and low income zip code could potentially bias our findings by not taking into account variability amongst all higher income zip codes and all lower income zip codes. The effect of this bias may be greatest when comparing findings between each county as a whole, as we only analyzed two zip codes from each county. To compare high and low income zip codes, however, we matched the zip codes by numerous demographic factors between each of the three counties to create an aggregate of high and low income zip codes. The exclusion of duplicate chain stores could have an effect on our results, potentially leading to a sample size from lower income zip codes that was twice that of high income zip codes. Nevertheless, the notable differences in sunscreens available by community demographics must raise further inquiry, as the supply of sunscreen could easily have an effect on consumer habits.

Though our study investigated 3 metropolitan counties, it can potentially extend beyond just large urban settings. In a study of sunscreen usage with planned exposure, rural residents were equally likely as urban residents to use sunscreen following the removal of confounding variables [45]. More research is needed to evaluate sales patterns within communities, and in areas outside of the large city setting to better understand the relationship between market availability and consumer habits and regional differences.

## 4. Conclusion

The market for sunscreens is constantly evolving, requiring further research to ensure the maintenance of adequate skin cancer and sunburn prevention. The study of changes in sunscreen patterns, however, requires an epidemiological approach, as community structure and regional differences noticeably alter the supply side of the sunscreen market. With this information, preventative education and initiatives can be tailored to individual community needs.

## Figures and Tables

**Table 1:** Demographics of selected zip codes.

	Cook County		San Diego County		Miami-Dade County	
Zip code	60611	60622	92127	92025	33156	33189
Median household income	$85,583	$64,037	$117,016	$45,998	$94,399	$51,677
Population	28,718	52,548	39,337	49,978	31,315	23,828
Median age	39.5	31.0	35.2	31.1	41.3	36.0
High school graduate or higher	99.5%	86.9%	96.6%	70.3%	93%	84%
Unemployed	6.7%	5.3%	5.4%	8.1%	5.4%	10.4%
Hispanic or Latino	4.9%	29.1%	9.5%	55.3%	47.9%	56.9%

**Table 2:** Melanoma incidence and demographic information of Miami-Dade, Cook, and San Diego Counties.

County	Melanoma incidence	Median household income^22^	High school degree or higher in 25+ year olds^22^	% White non-Hispanic^22^
	2006–2010	2007–2011	2007–2011	2012
Miami-Dade	8.7 per 100,000	$43,957	77.6%	16.3%
Cook	12.6 per 100,000	$54,598	83.7%	43.4%
San Diego	26.9 per 100,000	$63,857	85.3%	47.6%

**Table 3:** Makeup of the sampled sunscreen isles with comparisons between the price per ounce, corrected for SPF, of physical versus chemical sunscreens and spray versus lotion sunscreens.

Sunscreens sampled *n* = 1,660	
% Tanning oils	11.0%
% SPF < 15	14.9%
% SPF > 15	65.4%
% Physical sunscreens	3.4%
Price per ounce · SPF physical = $0.10	
Price per ounce · SPF chemical = $0.07	
*P* < 0.01	
% Spray sunscreens	46.2%
Price per ounce · SPF lotion = $0.08	
Price per ounce · SPF spray = $0.03	
*P* < 0.01	
Mean price per Oz.	$2.10
Mean corrected SPF	34.3

**Table 4:** Available sun products by zip code with comparisons between high and low income zip codes. “High income” zip codes represent the aggregates 92127, 33156, and 60611. “Low income” zip codes represent the aggregates 92025, 33189, and 60622.

	Zip code	Number of sunscreens sampled	Tanning oils %	SPF < 15%	SPF > 15%	Physical sunscreen %	Price per ounce	Spray %	Mean corrected SPF
Region	High income	552	16.3%	19.7%	59.6%	3.9%	1.97	40.3	30.8
	Lower income	1108	8.3%	12.2%	68.3%	3.1%	2.17	49.2	36.1
	*P* value		*P* < 0.01	*P* < 0.01	*P* < 0.01	*P* = 0.40	*P* = 0.03	*P* < 0.01	*P* < 0.01

San Diego County	92127	171	10.5%	20.5%	43.9%	4.6%	$2.12	40.5%	33.4
	92025	475	3.6%	15.4%	55.4%	3.3%	$2.25	48.7%	37.6
	*P* value		*P* < 0.01	*P* = 0.15	*P* = 0.01	*P* = 0.62	*P* = 0.03	*P* = 0.10	*P* < 0.01

Miami-Dade County	33156	259	23.9%	25.1%	59.5%	0.5%	$1.87	39.6%	26.3
	33189	404	15.1%	11.9%	71.8%	0.6%	$1.94	55.1%	32.6
	*P* value		*P* < 0.01	*P* < 0.01	*P* < 0.01	*P* = 0.70*	*P* = 0.01	*P* < 0.01	*P* < 0.01

Cook County	60611	122	8.2%	7.4%	82.0%	8.9%	$2.03	41.1%	36.6
	60622	229	6.1%	6.1%	89.1%	6.5%	$2.19	40.9%	39.3
	*P* value		*P* = 0.61	*P* = 0.82	*P* = 0.90	*P* = 0.53	*P* = 0.18	*P* = 0.92	*P* = 0.10

*Fischer-exact test used in place of *χ* ^2^.
